# Expression of genes for bone morphogenetic proteins BMP-2, BMP-4 and BMP-6 in various parts of the human skeleton

**DOI:** 10.1186/1471-2474-8-128

**Published:** 2007-12-27

**Authors:** Iwona Kochanowska, Slawomir Chaberek, Andrzej Wojtowicz, Bartosz Marczyński, Krzysztof Włodarski, Maria Dytko, Kazimierz Ostrowski

**Affiliations:** 1 Ludwik Hirszfeld Institute of Immunology and Experimental Therapy Polish Academy of Sciences, R. Weigla 12 St., Wroclaw, 53-114, Poland; 2 Department of Dental Surgery, Medical University, Chalubinskiego5, Warsaw, 02-006, Poland; 3 Department of Histology, Medical University, Chalubinskiego 5, Warsaw, 02-004, Poland; 4 Independent Orthopaedical Hospital, Konarskiego 13, Otwock, 05-400, Poland

## Abstract

**Background:**

Differences in duration of bone healing in various parts of the human skeleton are common experience for orthopaedic surgeons. The reason for these differences is not obvious and not clear.

**Methods:**

In this paper we decided to measure by the use of real-time RT-PCR technique the level of expression of genes for some isoforms of bone morphogenetic proteins (BMPs), whose role is proven in bone formation, bone induction and bone turnover. Seven bone samples recovered from various parts of skeletons from six cadavers of young healthy men who died in traffic accidents were collected. Activity of genes for BMP-2, -4 and -6 was measured by the use of fluorescent SYBR Green I.

**Results:**

It was found that expression of m-RNA for BMP-2 and BMP-4 is higher in trabecular bone in epiphyses of long bones, cranial flat bones and corpus mandibulae then in the compact bone of diaphyses of long bones. In all samples examined the expression of m-RNA for BMP-4 was higher than for BMP-2.

**Conclusion:**

It was shown that m-RNA for BMP-6 is not expressed in the collected samples at all. It is postulated that differences in the level of activation of genes for BMPs is one of the important factors which determine the differences in duration of bone healing of various parts of the human skeleton.

## Background

Bone mass is a changeable parameter. Its peak is reached in the age of 30–35 years. Bone turnover is under the control of two cell populations – osteoblasts and osteoclasts. These cells are influenced by many factors which control the balance of bone formation and bone resorption. The same cell populations and factors are active in bone healing. Bone morphogenetic proteins (BMPs) are involved in many processes which take place in bone formation, bone induction, and bone regeneration as e.g. callus formation in the course of bone healing. The full picture of bone turnover is not completed as yet, although many factors and interactions are well described. The complexity of bone turnover involves the long list of about 200 factors influencing each other in various physiological and/or pathological situations. PTGF (platelet derived growth factor) and TGF-β (transforming growth factor – beta) together with the BMPs are the most important factors in the process of bone healing. The new research [1] data make the situation even more complex.

Differences in the dynamic of bone healing in various parts of skeleton are well documented in literature. In population of healthy people the cortical bone heals within 4–8 months, and trabecular bone in 3–6 months. The healing of cranial bones can take from several weeks to 5 years. Broken mandible heals normally during 3–4 weeks. It takes several months for healing of broken bones of pelvis. Broken humeri are healed in 3–6 months. Similar time is needed for healing of femurs. Complexity of orthopaedic situation has important influence on time of bone repair [2-7].

BMPs play very important role in bone physiology influencing bone growth, turnover, bone formation and cartilage induction [8]. Their influence is not restricted to bone tissue. BMPs regulate apoptosis in various types of tissues [9,10]. It seems that the study of some BMPs isoforms reveals unexpected and very important activities of these proteins. In one of the recent publication by the group of Vescovi it was shown that the BMP-4 elicits strongest effect in triggering a significant reduction in tumor-initiating precursors of human glioblastoma [11]. All BMPs, except BMP-1, belong to the superfamily of transforming growth factors β, forming large group of signaling molecules [10,12-15]. Their activity is related only to dimeric form. Both homodimers as well as heterodimers are active [13,16], although their biological activity differs [14]. High activity of heterodimers BMP-4–7 and BMP-2–7 was established in vitro and in vivo [13]. High osteoinductive potency was proved for heterodimers BMP-2–6 in comparison with respective homodimers [17,18]. In bone matrix BMPs are connected with collagen and are not active unless released by the action of collagenases of osteoclasts [14].

According to the literature [19] BMPs -2, -4, -6 exert high osteoinductive potential, influencing odontogenesis [10,19-21], bone regeneration and healing [13,22-27], bone formation [15,17,28-31] and heterotopic bone induction [32-34]. In tissue culture BMPs are needed for formation of osteogenic cell lines [13,32,34,35].

The aim of this paper is to show that, in spite of incomplete knowledge of the mechanisms of bone homeostasis, some important differences exist at the molecular level, which can explain the differences in the dynamic of healing of distinct parts of human skeleton. By the use of quantitative real-time RT-PCR we were able to show the differences in the level of expression of some isoforms of BMPs in various bones or their parts.

The results were confirmed by the analysis of the electrophoretic bands.

## Methods

### Tissue samples and total RNA isolation and quantification

Forty two biopsies of normal bone tissue taken *post mortem *from six cadavers of young healthy men who died in traffic accidents were frozen. Biopsies were taken from seven different bones. The cadavers are treated as "young healthy" after the routine serology done for all tissue bank donors. Biopsies (5–7 cm long and 2–3 cm wide) were taken from seven different bones. Bone marrow was removed from trabecular bone by the routine technique used in our bone bank. There were: cranial flat bones, corpus mandibulae, diaphysis radii, distal epiphysis radii, ala ossis ilii, diaphysis femoris and distal epiphysis femoris. Total RNA was extracted using TRIzol Reagent (GIBCO BRL) according to manufacturer's recommendations. Frozen in liquid nitrogen and physically powdered bone (200 mg) was suspended in 1 ml of TRIzol Reagent, vortexed and incubated 30 min at room temperature with continuous horizontal rotation. The RNA pellet was dissolved in 20–30 μl of sterile diethylpyrocarbonate-treated Mili-Q water and quantified spectrophotometrically at 260 nm. The quality of the extracted RNAs was verified by agarose gel electrophoresis. RNAs were stored as a water solution at -70°C. The "donors" were transported after accidents to the Dept. of Forensic Medicine and located for the night in cold compartments. The samples were collected in the morning after medico-legal section and frozen in dry ice. We never attempted the investigation on degradation of beta-actin or BMPs – mRNAs as a function of time.

### Reverse transcription

Single stranded complementary DNA (cDNA) was synthesized with oligo(dT)_12–18 _primers from 5 μg of total RNA using SUPERSCRIPT First-Strand Synthesis System for RT-PCR (GIBCO BRL) accordingly to the manufacturer's instruction.

### Quantitation of BMPs expression by real-time PCR

Accumulation of PCR products was measured in real-time by using QuantiTect SYBR Green PCR Kit (Qiagen). The sequences of primers are listed in Table 1. Reaction was performed in DNA Engine Opticon^® ^2 Real-Time Detection System (MJ Research) three times for each probe starting with 10 – 15 min of preincubation at 94°C followed by 45 amplification cycles as follows: 94°C for 1 min, annealing temperature for 1 min and 72°C for 1 min. Beta-actin was used as housekeeping gene for arbitrary unit calculation for every tested gen. Additionally product identity was confirmed by electrophoresis on a 1.7% agarose (GIBCO BRL) gel and visualized by ethidium bromide (SIGMA) staining under UV light.

**Table 1:** Primer sequences and sizes of amplicons generated by Real-time RT-PCR

mRNA	Forward primer (5'-3')	Reverse primer (5'-3')	Annealing [°C]	Amplicon [bp]	Accession number	Citation
BMP-2	ATGGATTCGTGGTGGAAGTG	GTGGAGTTCAGATGATCAGC	58	349	NM_001200.2	[41]
BMP-4	AGCATGTCAGGATTAGCCGA	TGGAGATGGCACTCAGTTCA	58	399	NM_130851.1	[41]
BMP-6	CAGCCTGCAGGAAGCATGAG	CAAAGTAAAGAACCGAGATG	53	246	NM_001718.2	[42]
β-actin	GGGTCAGAAGGATTCCTATG	GGTCTCAAACATGATCTGGG	58	237	NM_001101.2	[43]

Statitistics: All statistical analysis was performed using the STATISTICA 5.0 software (StatSoft, Inc., STATISTICA for Windows, Tulsa, OK). We compared the analysed groups using the analysis of variance (ANOVA). Levels of statistical significance was set as p = 0,05: S-significant difference (p < = 0,05), NS – no statistical difference was found (p > 0,05).

## Results

**Figure 1:**
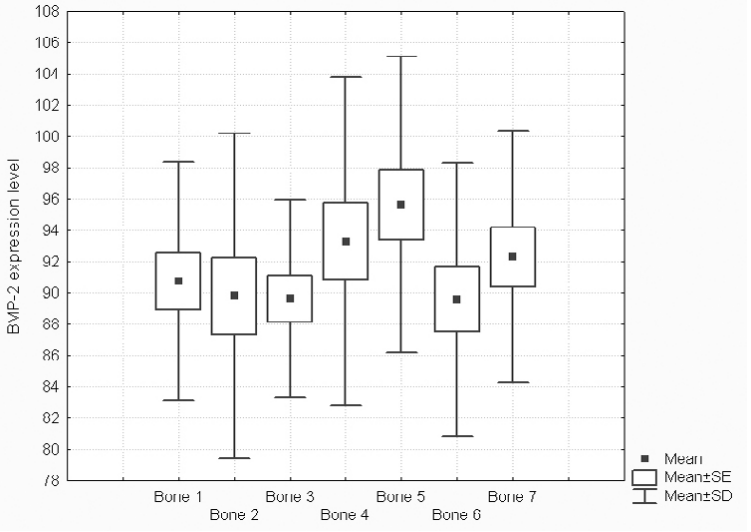
Comparison of BMP-2 expression levels in separate bone groups. Box plot graph, Mean, SE – Standard error, SD – Standard Deviation.

**Figure 2:**
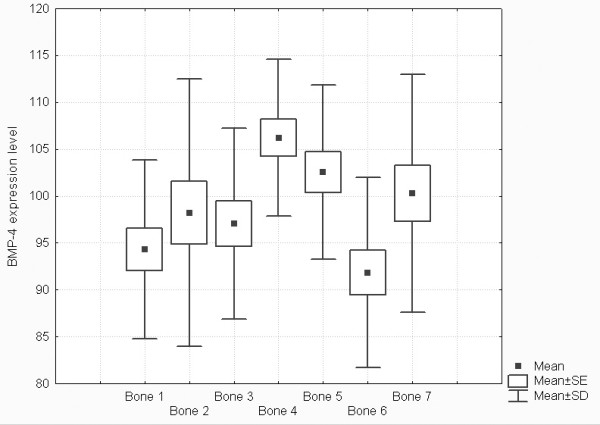
Comparison of BMP-4 expression levels in separate bone groups. Box plot graph, Mean, SE – Standard error, SD – Standard Deviation.

**Figure 3:**
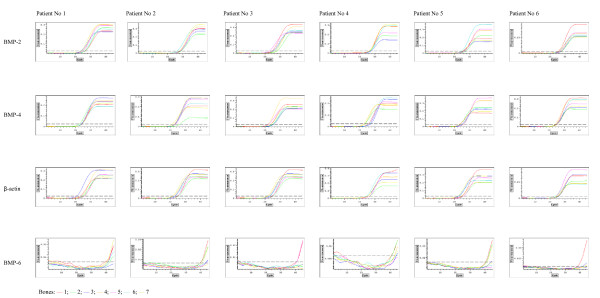
Expression of BMPs in different bone biopsies derived from 6 patients quantified by real-time PCR.

**Figure 4:**
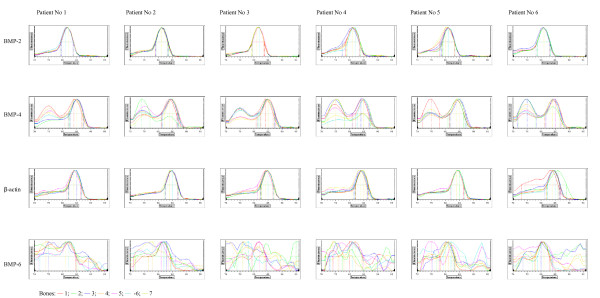
Melting curves of real-time PCR products.

**Figure 5:**
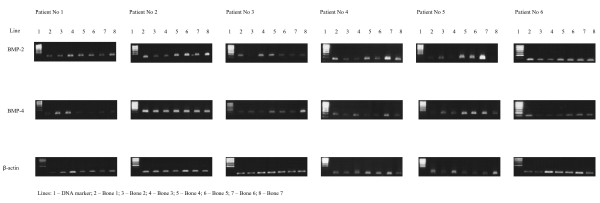
Electrophoretic analysis of real-time PCR products.

Real-time PCR technique was very useful for detection, determination and comparison of three BMP isoforms expressions. The use of β-actin gene expression as a housekeeping gene allowed for calculation of arbitrary units and for comparison of BMP-2, BMP-4 and BMP-6 genes expression not only between them but also between different types of bones. Obtained results showed significant expression of two isoforms of these proteins – BMP-2 and BMP-4 (Figures 1 and 2, respectively). Expression of BMP-6 was not detected. Melting temperatures of amplicons demonstrate their specifity (Figure 3) and electrophoretic analysis proved identity of these products (Figures 4 and 5). This multi-analysis allowed for conclusions that there is no BMP-6 gene expression in all examined bone samples.

## Discussion

In this paper we demonstrated the differences in expression of m-RNA for isoforms BMP-2, BMP-4 in various parts of the human skeleton (Table 2).

**Table 2:** The number and codes of examined skeletal bone samples

Group Name	Group Code	N	
		BMP – 2	BMP – 4

Cranial flat bones	Bone 1	18	18
Corpus mandibulae	Bone 2	18	18
Diaphysis radii	Bone 3	18	18
Distal epiphysis radii	Bone 4	18	18
Ala ossis ilii	Bone 5	18	18
Diaphysis femoris	Bone 6	18	18
Distal epiphysis femoris	Bone 7	18	18

These differences concerned expression of m-RNAs for genes BMP-2 and BMP-4. In the same bone sample the level of expression of m-RNAs for BMP-2 and BMP-4 differ. These differences are significant when compact bone and trabecular bone are compared.

Results of descriptive statistics (Fig 1) show that expression of m-RNA for BMP-2 is higher in ala ossis illi, and in trabecular bone of epiphyses of long bones as well as of flat bones then in corpus mandibulae and in the compact bone of diaphyses of long bones (Fig 1). The level of expression of BMP-4 is higher in trabecular bone in epiphyses of long bones, ala ossis illi and corpus mandibulae then in the compact bone of diaphyses of long bones and cranial flat bones (Fig 2).

In all examined samples the level of expression of m-RNA for BMP-4 was higher than for BMP-2 (Fig. 1 and Fig. 2).

Several repetitions of measurements by real-time RT-PCR excluded expression of mRNA for BMP-6 in analysed samples.

Our results differ from the ones which were published by the Thailand group [36]. We are not fully convinced by their results. They used the semiquantitative technique of RT-PCR and the measurements were based on the intensity of fluorescence of the agarose gels standardized against cDNA for control gene for GAPDH (glyceraldehyde-3-phosphate dehydrogenase). Their samples were taken from patients in the course of surgery and it is difficult to call them "normal control samples". In the healing bones after fracture, activation of BMP-6 gene is expected. Literature data [28,37,38] allow us to assume, that the lack of m-RNA for BMP-6 in all our samples was compensated by activity of BMP-2 [28]. Because of the high osteogenic activity of homodimers BMP-2/BMP-2 and BMP-4/BMP-4 and of high probability of formation of heterodimers BMP-2/BMP-4, the lack of BMP-6 can be explained by the substitution by homodimers BMP-2/BMP-4. The additional argument for our reasoning is the high identity in sequence of proteins BMP-2 and BMP-4 reaching 92% [10,13,39,40]. Both belong to the same subclass, in contrast to BMP-6, which is the member of different subfamily.

## Conclusion

The analysis of various parts of skeleton of six healthy men who died in traffic accidents showed several differences in expression of some BMPs- isoforms as measured by real-time RT-PCR spectrometry:

1. Various bones forming the skeleton differ in the level of expression of BMP-2 and BMP-4 isoforms.

2. Significant differences were found in expression of BMP-2 between cranial flat bones and ala ossis ilii, corpus mandibulae and ala ossis ilii, diaphysis radii and ala ossis ilii, diaphysis femoris and distal epiphysis femoris (Table 3, Figure 1)

**Table 3:** Evaluation of significant differences between mean levels of BMP-2 expression in all examined bones. S – significant difference (p < = 0,05); NS – no statistical difference was found (p > 0,05)

	Bone 1	Bone 2	Bone 3	Bone 4	Bone 5	Bone 6	Bone 7
Bone 1	-	NS	NS	NS	S	NS	NS
Bone 2	NS	-	NS	NS	S	NS	NS
Bone 3	NS	NS	-	NS	S	NS	NS
Bone 4	NS	NS	NS	-	NS	NS	NS
Bone 5	S	S	S	NS	-	S	NS
Bone 6	NS	NS	NS	NS	S	-	NS
Bone 7	NS	NS	NS	NS	NS	NS	-

3. Significant differences were found in expression of BMP-4 between cranial flat bones and ala ossis ilii, cranial flat bones and distal epiphysis radii, corpus mandibulae and distal epiphysis radii, diaphysis and distal epiphysis radii, diaphysis radii and ala ossis ilii, diaphysis radii and ala ossis ilii, distal epiphysis radii and ala ossis ilii, distal epiphysis radii and diaphysis femoris, ala ossis ilii and diaphysis femoris, diaphysis and distal epiphysis femoris. (Table 4, Figure 2).

**Table 4:** Evaluation of significant differences between mean levels of BMP-4 expression in all examined bones. S – significant difference (p < = 0,05); NS – no statistical difference was found (p > 0,05)

	Bone 1	Bone 2	Bone 3	Bone 4	Bone 5	Bone 6	Bone 7
Bone 1	-	NS	NS	S	S	NS	NS
Bone 2	NS	-	NS	S	NS	NS	NS
Bone 3	NS	NS	-	S	S	NS	NS
Bone 4	S	S	S	-	S	S	NS
Bone 5	S	NS	S	S	-	S	NS
Bone 6	NS	NS	NS	S	S	-	S
Bone 7	NS	NS	NS	NS	NS	S	-

4. Significant differences exist in the mean values of the levels of expression of BMP-2 when compared with the level of BMP-4 between the following parts of skeletons: diaphysis radii, distal epiphysis radii, ala ossis ilii, and distal epiphysis femoris (Table 5).

**Table 5:** The results of the comparison by the Analysis of Variance (ANOVA) (p = 0,05) of the mean levels of expression of BMP-2 and the levels of BMP-4 in the groups of examined bones. S – significant difference (p < = 0,05); NS – no statistical difference was found (p > 0,05)

		Mean BMP-2	Mean BMP-4	Std. Dev. BMP-2	Std. Dev. BMP-4	Valid N BMP-2	Valid N BMP-4	F	p
Bone 1	NS	90,7208	94,3128	7,6504	9,4994	18	18	1,5610	0,2200
Bone 2	NS	89,7981	98,2081	10,4187	14,2636	18	18	4,0804	0,0513
Bone 3	S	89,6294	97,0668	6,2952	10,1941	18	18	6,9357	0,0126
Bone 4	S	93,3007	106,2281	10,4861	8,3981	18	18	16,6666	0,0002
Bone 5	S	95,6507	102,5552	9,4378	9,2962	18	18	4,8896	0,0338
Bone 6	NS	89,5953	91,8471	8,7169	10,1045	18	18	0,5124	0,4789
Bone 7	S	92,2981	100,2836	8,0808	12,6990	18	18	5,0663	0,0309

5. In all other measured parts of skeleton the levels of expression of the two isoforms of BMP were similar and did not differ significantly.

6. The expression of BMP-6 isoform was not found in all examined samples.

The obtained results might help to understand the differences in the speed of healing of various parts of the skeleton. We have started the measurements of samples of callus obtained in the course of surgical revisions of slow healing bone fractures.

The obtained results might also have some value for the bone banks where the level of expression of BMPs could change after single steps of bone preservation as freezing, liophilisation, radiation sterilization etc. The expected role of bone implants prepared in bone banks is the promotion of bone healing by two processes – conduction and induction.

The process of bone induction in human orthopaedic practice is not well documented.

Some bone banks try to promote this ability by adding synthetic recombined BMPs to bone implants, sometimes with the addition of hydroxyapatite. This is why the knowledge of bioinductive properties of single parts of skeleton is important.

## Competing interests

The author(s) declare that they have no competing interests.

## Authors' contributions

All authors contributed equally to this work

## Pre-publication history

The pre-publication history for this paper can be accessed here:


